# Factor structure and reliability of the symptom measurement of post-stroke depression in the rehabilitation stage

**DOI:** 10.1186/s12888-024-05906-w

**Published:** 2024-06-14

**Authors:** Yawei Zeng, Junya Chen, Jing Liu, Yi Zhang, Hongxia Wang, Yanhong Jiang, Weiwei Ding, Yun Li, Jufang Li

**Affiliations:** 1 School of Nursing, Wenzhou Medical University, Wenzhou, Zhejiang China; 2 The First Affiliated Hospital of Wenzhou Medical University, Wenzhou, Zhejiang China

**Keywords:** Post-stroke depression, Stroke patient, Exploratory factor analysis, Confirmatory factor analysis, Measurement development, Rehabilitation stage

## Abstract

**Background:**

The incidence of Post Stroke Depression (PSD) in the Rehabilitation Stage is high, which can bring serious physical and psychological disorders to patients. However, there is still a lack of targeted tools for screening PSD in the rehabilitation stage. Therefore, the aim of this study was to evaluate the factor structure and reliability of a measurement instrument to screen for PSD in the rehabilitation stage.

**Methods:**

A cross-sectional study was conducted on 780 hospitalized stroke patients who were within the rehabilitation stage from May to August 2020. Exploratory factor analysis (EFA) as well as first- and second-order confirmatory factor analysis (CFA) were performed to evaluate the factor structure of the newly developed Symptom Measurement of Post-Stroke Depression in the Rehabilitation Stage (SMPSD-RS). The reliability and validity of the SMPSD-RS were also verified using several statistical methods.

**Results:**

EFA extracted a 24-item, five-factor (cognition, sleep, behavior, emotion, and obsession) model that can clinically explain the symptoms of PSD during the rehabilitation stage. A first-order CFA confirmed the EFA model with good model fit indices, and the second-order CFA further confirmed the five-factor structure model and showed acceptable model fit indices. Acceptable reliability and validity were also achieved by the corresponding indicators.

**Conclusion:**

The SMPSD-RS was proven to have a stable factor structure and was confirmed to be reliable and valid for assessing PSD symptoms in stroke patients during the rehabilitation stage.

## Introduction

Post-stroke depression (PSD) is one of the most frequent mental health complications following stroke, and mostly presents as depressed mood, anhedonia, apathy syndrome, insomnia, fatigue, and amnestic disorder [1–3]. PSD exists in about one third of stroke patients at any stage following stroke and is related to increased mortality among stroke survivors [4]. However, some studies have pointed out that the incidence of PSD in the rehabilitation stage is higher than that in the early and sequelae stages [5]. PSD in the rehabilitation stage refers to depressive symptoms that occur within one and six months after stroke [6], which is mainly characterized by increased dependence, insufficient enthusiasm for rehabilitation treatment, and insufficient confidence in rehabilitation outcomes [7]. PSD in the rehabilitation stage is related to lower levels of social support, higher levels of physical and cognitive functional impairment, and uncontrollable feelings about rehabilitation outcomes [8, 9]. It is further associated with lower quality of life [3], leads to reduced independence in daily life and affects the subsequent recovery of stroke patients [10]. Due to the above reasons, routine screening of PSD symptoms in the rehabilitation stage is recommended [11], which will enable the development of targeted intervention strategies that will promote the functional recovery and social integration of stroke patients.

At present, three types of screening tools are used to assess PSD in the rehabilitation stage: the Diagnostic and Statistical Manual of Mental Disorders-V (DSM-V), rating scales used for assessing general depression, and rating scales specific to PSD. However, all these instruments have certain shortcomings when screening for PSD in the rehabilitation stage. Specifically, the DSM-V requires professional participation and takes a long time; thus, it is not suitable for routine PSD screening in clinical settings. The rating scales used for assessing general depression that are also used to assess PSD in the rehabilitation stage, such as the Hamilton Depression Rating Scale [12], the Beck Depression Inventory II [13], the Montgomery and Asberg Depression Rating Scale [14], the Geriatric Depression Scale [15], and the Patient Health Questionnaire (PHQ-9) [16] are not designed for use with the Chinese population and are not specifically designed for stroke patients [17]; therefore, they lack sensitivity and specificity when used with Chinese stroke patients. Notably, as one of the rating scales used for assessing general depression, PHQ-9 appears to be the optimal and pragmatic screening tool for PSD at present [4]. Therefore, PHQ-9 was used in this study to test the concurrent criterion validity of the SMPSD-RS. Rating scales specific to PSD such as the Post-Stroke Depression Rating Scale (PSDRS) [18], the Yale-Brown Single Item Screening Question [19], the Post-Stroke Depression Scale (PSDS) [20], and the Early Symptom Measurement of Post-Stroke Depression (ESM-PSD) [21] have also been shown to be problematic when used to screen for PSD in the rehabilitation stage. The screening results of the PSDRS are greatly affected by age [22]. Since the Yale-Brown Single Item Screening Question consists of only one question, it is unable to fully describe PSD symptoms in the rehabilitation stage. The sample size used in the development of the PSDS was inadequate (158 cases), meaning that further testing is needed to verify its reliability, validity, and screening efficacy [20]. The ESM-PSD is specifically desiged for screening PSD in the early stage of stroke [21], which may not suitable for screening PSD in the rehabilitation stage. Due to the shortcomings of the current tools for assessing PSD in the rehabilitation stage and the lack of a screening tool specifically developed to assess PSD symptoms in the rehabilitation stage after stroke, it was a good idea to develop a measurement instrument to screen for PSD in the rehabilitation stage [17]. The present research aimed to test the psychometric properties of the Symptom Measurement of Post-Stroke Depression in the Rehabilitation Stage (SMPSD-RS), a new scale specifically developed to screen for PSD in Chinese stroke patients who are in the rehabilitation stage [23].

## Methods

### Sample

Purposive sampling was used to recruit participants from the rehabilitation department of a general hospital in Southeast China from May to August 2020. Specifically, participants were recruited during their inpatient period. The inclusion criteria were as follows: patients whose stroke diagnosis had been confirmed by computed tomography or magnetic resonance imaging, with stable vital signs, mental clarity, a timeframe of between one and six months after stroke, and who could communicate in either written or verbal form. Participants with subarachnoid hemorrhage, serious heart, liver, and renal insufficiency, cancer, loss of consciousness, sensory aphasia, or cognitive impairment were excluded. The study’s sample size was determined based on the statistical methods we used. Some researchers suggest that when performing exploratory factor analysis (EFA), the expected number of participants should be between five and ten times the number of items on the questionnaire (Rouquette & Falissard, 2011). Since SMPSD-RS consists of thirty-three items, the required sample size for EFA would be between 165 and 330. Assuming that 20% of the questionnaires are invalid, between 207 and 413 participants would be required. In addition, the sample size required for confirmatory factor analysis (CFA) should be between five and ten times the freely estimated parameters in the CFA. As the number of freely estimated parameters in the CFA could not be determined until data analysis, the research team determined the sample size of the CFA to be greater than that of the EFA. Among the 807 participants approached by the researchers, twelve chose not to participate and fifteen withdrew during the investigation due to physical discomfort. Finally, 780 participants completed the survey and their data were eligible for analysis. The PHQ-9 was distributed to thirty participants who were randomly selected from the 780 participants to test the concurrent criterion validity of the SMPSD-RS. And the SMPSD-RS was distributed two weeks later to 50 participants who were also randomly selected from the 780 participants to evaluate the test-retest reliability of the SMPSD-RS.

### Instrument

#### Demographic and clinical status

A demographic and clinical characteristics questionnaire was compiled based on a literature review [24]. Demographic characteristics included age, sex, marital status, educational level, monthly household income, family relationship, living alone or with family, working status before stroke, place of residence, religious beliefs, medical payment method, and primary caregivers. Clinical status included days after stroke, the sleep hours per day, number of strokes, ability to walk on their own, incontinence and type of stroke.

#### The SMPSD-RS

The SMPSD-RS is developed by our research team based on the Cannon-Bard theory of emotion and through the Delphi method, which is specific for the identification of PSD in the rehabilitation stage. The SMPSD-RS includes 33 items with 6 dimensions (cognition, sleep, behavior, emotion, body, and guilt). The SMPSD-RS showed acceptable content validity as evidenced by the following indicators: the item-level content validity index = 0.780–1.000, the scale-level content validity index/universal agreement = 0.610, and the scale-level content validity index/ average = 0.970 [23]. Participants were required to complete the survey based on the frequency of their symptoms during the previous week. The self-rating SMPSD-RS is scored on a 5-point Likert scale (0 = *never*, 1 = *occasionally*, 2 = *sometimes*, 3 = *often*, 4 = *always*). The total score of the scale is determined by adding up the score for each item. A higher total score indicates a higher degree of depression.

#### The PHQ-9

The PHQ-9 assesses depression in two domains, namely the somatic domain and the cognitive domain [25]. It consists of nine items on a four-point Likert scale. The somatic domain is scored by five items (3, 4, 5, 7, and 8) and the cognitive domain is scored by four items (1, 2, 6, and 9). Studies have shown that the PHQ-9 has a good screening effect for PSD patients, with high sensitivity and high accuracy [26]. Therefore, the PHQ-9 was used as the gold standard to test the concurrent criterion validity of the SMPSD-RS. The Cronbach’s α for the PHQ-9 in stroke patients was 0.892 in one previous study [27], and the Cronbach’s α for the PHQ-9 in this study was 0.871.

### Data collection

Potential participants were identified through the electronic medical record system of the hospital. Prospective participants were approached by the research team members and informed of the study purpose. Thereafter, a 30-minute private face-to-face interview was conducted with those participants who had signed the written informed consent form, and they were assured that they were free to withdraw if they felt unwell. Face-to-face interviews were conducted in Mandarin, and the participants were invited to complete the questionnaire themselves or, if they had difficulty doing so, the researchers could record the answers on their behalf.

### Data analysis

Data were analyzed using SPSS 26.0 and MPLUS 8.0. Statistical significance was set at *p* < 0.05. Descriptive analysis was employed to depict the participants’ demographic and clinical characteristics. Specifically, frequencies and percentages were employed to report categorical variables and means and standard deviations were employed to report continuous variables.

EFA and CFA were conducted to explore the factor structure of the SMPSD-RS. The sample of 780 participants was randomly divided into two data sets using the SPSS Select Cases option, and no significant difference was found between the two data sets. An EFA was run on the first data set of 385 participants. Bartlett’s test of sphericity and the Kaiser-Meyer-Olkin (KMO) index were used to confirm sample suitability for EFA, with a KMO measure greater than 0.600 indicating that the sample was suitable for EFA [28, 29]. Principal component analysis and varimax rotation were used to run the EFA [30]. Items with factor loadings of 0.350 or higher were retained [31], and factors with eigenvalues greater than 1.000 were retained. The selected items should explain at least 5% of the total variance of PSD in each factor, and all selected factors should explain at least 60% of the total variance of PSD for the whole measurement [31]. The second data set comprising 395 participants was subjected to CFA to confirm the factor structure suggested by the EFA. Item factor loadings in the CFA should be greater than or equal to 0.500 [32]. The goodness of fit indices of the CFA were set as follows: χ^2^/*df* less than 5.000, comparative fit index (CFI) greater than 0.900, Tucker-Lewis index (TLI) greater than 0.900, root mean square error of approximation (RMSEA) less than 0.080, and standardized root mean square residual (SRMR) less than 0.080 [33, 34]. A second-order CFA was also employed to evaluate the level of contribution of all factors extracted by the EFA to symptoms of PSD in the rehabilitation stage.

The internal reliability of SMPSD-RS was evaluated using internal consistency indicators, such as Cronbach’s α, corrected item-total correlation, item-subscale correlation, and composite reliability [21]. Acceptable internal reliability was defined as Cronbach’s α greater than or equal to 0.700, corrected item-total correlation greater than or equal to 0.400, item-subscale correlation greater than or equal to 0.400, and composite reliability greater than or equal to 0.700 [35]. In addition, the intraclass correlation coefficient (ICC) was employed to assess the test-retest reliability [36]. An ICC between 0.750 and 0.900 demonstrates good reliability, and an ICC greater than 0.900 demonstrates superior reliability [37].

The discriminant validity of the SMPSD-RS was assessed by the average variance extracted (AVE), which was required to be greater or equal to 0.500 [38]. Acceptable evidence for discriminant validity is also confirmed if the AVE’s square root value belonging to each potential domain is greater than the correlation between any pair of potential domains [39]. In addition, acceptable concurrent criterion validity was evidenced by a significant Pearson correlation coefficient of 0.400–0.800 between the SMPSD-RS and the PHQ-9 [40].

#### Ethical considerations

This study was approved by the Ethics Committee of the First Affiliated Hospital of Wenzhou Medical University (Approval number: 2020-zz-072). Before the formal investigation, the researcher introduced the content and purpose of the study to the respondents, informed the participants that they could voluntarily choose to participate in the study or not, and informed them that they could quit the study at any time during the study. The survey was conducted after obtaining informed consent, and the data obtained from the survey was only used for this study. We confirm that all the methods used in this study were carried out in accordance with the guidelines and provisions of the Declaration of Helsinki.

## Results

### Participants’ demographic and clinical characteristics

Participants’ demographic and clinical characteristics are shown in Table 1. The average age of the participants were 63.05 (*SD* = 12.04) and 65.22 (SD = 12.62) for the EFA and CFA sample. In addition, most of the participants were married (87.27% for the EFA sample and 92.15% for the CFA sample), male (69.87% for the EFA sample and 66.84% for the CFA sample), and had more than six years of education (65.45% for the EFA sample and 68.61% for the CFA sample). Please refer to Table 1 for other demographic and clinical information.

**Table 1:** Demographic and clinical characteristics of the participants (*N* = 780)

Variables	EFA sample = 385	%	CFA sample = 395	%
**Demographic characteristics**				
Age (years)	63.05 (*M*)	12.04 (*SD*)	65.22 *(M)*	12.62*(SD)*
Male	269	69.87	264	66.84
Married	336	87.27	364	92.15
Educated	252	65.45	271	68.61
High monthly household income^a^	145	37.66	122	30.89
Family relationship: harmonious	383	99.48	392	99.24
Living with families	365	94.81	375	94.94
Employed and farmers	219	56.88	183	46.33
Place of residence: rural	210	54.55	259	65.57
No religious belief	250	64.94	254	64.30
Rural cooperative medical service	248	64.42	274	69.37
Primary Caregiver: family members	259	67.27	240	60.76
**Clinical characteristics**				
Days after stroke	122.56 (*M*)	49.51 (*SD*)	127.70 *(M)*	30.39 *(SD)*
Sleep hours per day	7.09 (*M*)	1.17 (*SD*)	6.81 *(M)*	0.54 *(SD)*
First-time stroke	348	90.39	345	87.34
Walk on their own	238	61.82	297	75.19
Urinary incontinence	12	3.12	7	1.77
Type of stroke: cerebral infarction	262	68.05	305	77.22

EFA: exploratory factor analysis; CFA: confirmatory factor analysis; ^a^Chinese currency

### *The EFA and CFA of the SMPSD-RS*

Results pertaining to the EFA are shown in Table 2. A preliminary EFA with varimax rotation and principal component analysis suggested deleting nine items with extremely low factor loadings (items 5, 21, 23, 24, 29, 30, 31, 32, and 33). Thus, the number of items comprising the SMPSD-RS was reduced from 33 to 24. The KMO result for the 24-item scale was 0.950, and Bartlett’s test of sphericity (χ2 = 25095.943, *p* < 0.001) of the 385 participants confirmed the suitability for factor analysis. An EFA with the remaining 24 items extracted five factors with eigenvalues greater than 1. The five-factor structure model demonstrated good model fit: χ^2^/*df* = 2.750, CFI = 0.952, TLI = 0.920, RMSEA = 0.068, and SRMR = 0.028, which met our clinical interpretability and parsimony standards and were labeled as “emotion,” “behavior,” “sleep,” “cognition,” and “obsession.” The factor loadings of each item were met the required criteria of greater than 0.350. The variances explained by the five factors were 21.342%, 17.539%, 16.785%, 14.265%, and 11.741% for the domains of emotion, sleep, behavior, cognition, and obsession, respectively. The total variance explained by the five factors was 81.673%.

**Table 2:** Exploratory factor analysis of the SMPSD-RS (*N* = 385)

Items	Factors				
	Emotion	Behavior	Sleep	Cognition	Obsession
16. I can’t adjust my emotions.	**0.851**	0.219	0.202	0.204	0.197
15. I feel irritable.	**0.847**	0.143	0.241	0.210	0.222
18. I am emotional.	**0.820**	0.196	0.238	0.177	0.285
19. I blame others for trifles.	**0.812**	0.190	0.271	0.202	0.232
14. I feel depressed.	**0.661**	0.360	0.283	0.261	0.201
20. I lose interest in my surroundings.	**0.585**	0.482	0.197	0.257	0.230
12. I am unwilling to participate in the formulation of rehabilitation plans.	0.128	**0.752**	0.114	0.127	0.355
10. I am unable to initiate rehabilitation.	0.200	**0.725**	0.155	0.339	0.044
11. I depend on other’s in daily life.	0.114	**0.701**	0.187	0.176	0.408
13. I am not willing to communicate.	0.161	**0.686**	0.138	0.241	0.182
17. I want to cry or have cried.	0.347	**0.627**	0.148	-0.007	-0.046
6. I take longer to fall asleep.	0.235	0.197	**0.863**	0.165	0.130
7. I awaken easily.	0.270	0.175	**0.862**	0.178	0.154
8. I wake up early and then can’t fall asleep again.	0.246	0.172	**0.841**	0.178	0.185
9. I feel I am not getting enough sleep.	0.254	0.185	**0.697**	0.317	0.247
2. My thinking is not as clear as before.	0.217	0.221	0.203	**0.820**	0.280
1. My memory is worse than before (e.g., I can’t remember what was for breakfast).	0.262	0.230	0.260	**0.816**	0.240
3. I have difficulty concentrating.	0.297	0.219	0.260	**0.806**	0.245
4. I speak less than before.	0.202	0.538	0.201	**0.580**	0.039
25. I feel a malaise.	0.267	0.208	0.151	0.220	**0.753**
22. I feel too tired to do things.	0.298	0.088	0.215	0.280	**0.715**
28. I feel that stroke interfered with the work of my family members.	0.455	0.433	0.310	0.199	**0.564**
26. I feel that stroke increased the financial burden on my family.	0.454	0.423	0.305	0.204	**0.564**
27. I feel that stroke diminished my quality of life.	0.454	0.429	0.316	0.203	**0.560**
Eigenvalues	5.122	4.209	4.028	3.424	2.818
Variance explained by each factor (%)	21.342	17.539	16.785	14.265	11.741
χ2/*df*	2.750				
RMSEA	0.068 (0.061–0.075)				
CFI	0.952				
TLI	0.920				
SRMR	0.028				
**Items not loading on or not significant on any factor**					
5. I feel I have lost myself (such as life, family, etc.).					
21. I can’t sleep because of thinking a lot.					
23. I feel pain.					
24. I feel desperate in rehabilitation.					
29. I feel inability.					
30. I blame myself for past bad living habits.					
31. I blame myself for trifles.					
32. I have no confidence in rehabilitation.					
33. I feel that people like me deserve to die.					

SMPSD-RS: the Symptom Measurement of Post-Stroke Depression in the Rehabilitation Stage; Bold indicates the items loaded on each factor

The first-order CFA with the remaining 24 items demonstrated good model fit indices: χ^2^/*df* = 2.840, CFI = 0.945, TLI = 0.937, RMSEA = 0.068, and SRMR = 0.075, and the range of the CFA factor loadings was 0.506–0.997 (Table 3: First-order model). The second-order CFA with the five factors also demonstrated satisfactory model fit indices: χ^2^/*df* = 2.859, CFI = 0.943, TLI = 0.936, RMSEA = 0.069, and SRMR = 0.077 (Table 3: Second-order model), and the range of the CFA factor loadings was 0.730–0.885. The above statistical results confirmed the five-factor model of the SMPSD-RS.

**Table 3:** Confirmatory factor analysis of the SMPSD-RS (*N* = 395)

Variables	First-order model			Second-order model		
	SFL	SE	*p*	SFL	SE	*p*
**Factor 1 (Cognition)**				0.781	0.029	0.000
1. My memory is worse than before (e.g., I can’t remember what was for breakfast).	0.928	0.011	0.000			
2. My thinking is not as clear as before.	0.961	0.011	0.000			
3. I have difficulty concentrating.	0.946	0.008	0.000			
4. I speak less than before.	0.721	0.030	0.000			
**Factor 2 (Sleep)**				0.730	0.031	0.000
6. I take longer to fall asleep.	0.899	0.017	0.000			
7. I awaken easily.	0.940	0.013	0.000			
8. I wake up early and then can’t fall asleep again.	0.888	0.021	0.000			
9. I feel I am not getting enough sleep.	0.793	0.028	0.000			
**Factor 3 (Behavior)**				0.759	0.034	0.000
10. I am unable to initiate rehabilitation.	0.782	0.029	0.000			
11. I depend on other’s in daily life.	0.813	0.026	0.000			
12. I am unwilling to participate in the formulation of rehabilitation plans.	0.880	0.018	0.000			
13. I am not willing to communicate.	0.740	0.030	0.000			
17. I want to cry or have cried.	0.506	0.058	0.000			
**Factor 4 (Emotion)**				0.863	0.021	0.000
14. I feel depressed.	0.806	0.023	0.000			
15. I feel irritable.	0.902	0.015	0.000			
16. I can’t adjust my emotions.	0.883	0.020	0.000			
18. I am emotional.	0.950	0.011	0.000			
19. I blame others for trifles.	0.946	0.009	0.000			
20. I lose interest in my surroundings.	0.793	0.022	0.000			
**Factor 5 (Obsession)**				0.885	0.018	0.000
22. I feel too tired to do things.	0.583	0.035	0.000			
25. I feel a malaise.	0.645	0.036	0.000			
26. I feel that stroke increased the financial burden on my family.	0.990	0.004	0.000			
27. I feel that stroke diminished my quality of life.	0.997	0.001	0.000			
28. I feel that stroke interfered with the work of my family members.	0.988	0.008	0.000			
χ2/df	2.840			2.859		
CFI/ TLI	0.945/0.937			0.943/0.936		
RMSEA (90% CI)	0.068 (0.062–0.074)			0.069 (0.063–0.075)		
SRMR	0.075			0.077		

SFL: Standardized Factor Loading; SE: Standard Error; SMPSD-RS: the Symptom Measurement of Post-Stroke Depression in the Rehabilitation Stage

### The reliability and validity of the SMPSD-RS

Internal reliability evidence, such as Cronbach’s α, corrected item-total correlation, item-subscale correlation, and composite reliability were all acceptable. And All the ICC values were acceptable evidence for good test-retest reliability (Table 4).

**Table 4:** Cronbach’s alpha, corrected item-total correlation, item-subscale correlation, composite reliability and test-retest reliability of the SMPSD-RS (*N* = 395)

Items	Cronbach’s alpha	Corrected item-total correlation	Item-subscale correlation	Composite reliability	ICC (95% CI) (*N* = 50)
**Factor 1 (Cognition)**	0.936			0.940	0.849 (0.688–0.931)
1. My memory is worse than before ( e.g., I can’t remember what was for breakfast).		0.745	0.884		
2. My thinking is not as clear as before.		0.769	0.921		
3. I have difficulty concentrating.		0.791	0.909		
4. I speak less than before.		0.705	0.692		
**Factor 2 (Sleep)**	0.926			0.933	0.878 (0.744–0.944)
6. I take longer to fall asleep.		0.695	0.861		
7. I awaken easily.		0.691	0.893		
8. I wake up early then can’t fall asleep again.		0.641	0.832		
9. I feel I am not getting enough sleep.		0.705	0.746		
**Factor 3 (Behavior)**	0.859			0.866	0.896 (0.778–0.965)
10. I am unable to initiate rehabilitation.		0.589	0.731		
11. I depend on other’s in daily life.		0.650	0.714		
12. I am unwilling to participate in the formulation of rehabilitation plans.		0.649	0.817		
13. I am not willing to communicate.		0.639	0.690		
17. I want to cry or have cried.		0.479	0.470		
**Factor 4 (Emotion)**	0.955			0.954	0.923 (0.833–0.965)
14. I feel depressed.		0.787	0.802		
15. I feel irritable.		0.772	0.893		
16. I can’t adjust my emotions.		0.768	0.890		
18. I am emotional.		0.817	0.910		
19. I blame others for trifles.		0.819	0.905		
20. I lose interest in my surroundings.		0.789	0.767		
**Factor 5 (Obsession)**	0.926			0.932	0.921 (0.830–0.964)
22. I feel too tired to do things.		0.677	0.668		
25. I feel a malaise.		0.690	0.729		
26. I feel that stroke increased the financial burden on my family.		0.856	0.896		
27. I feel that stroke diminished my quality of life.		0.855	0.894		
28. I feel that stroke interfered with the work of my family members.		0.857	0.901		
**Total Scale**	0.967			0.985	0.973 (0.941–0.988)

*Note* SMPSD-RS: the Symptom Measurement of Post-Stroke Depression in the Rehabilitation Stage; ICC: Intraclass correlation coefficient; CI = Confidence Interval; **p **≤ 0.01**

The AVEs of the factors were 0.570–0.800, and the square roots of the AVEs were 0.755–0.894 (Table 5). The AVEs were all exceeded the standard value and the square roots of the AVEs of each individual domain were greater than the domain correlations, which were evidence of good discriminant validity of the SMPSD-RS. Besides, all the Pearson correlation coefficients of the SMPSD-RS and the PHQ-9 total and domain scores fell within the range of 0.400 and 0.800 (Table 6), which was evidence of good concurrent criterion validity of the SMPSD-RS.

**Table 5:** Estimated correlations between domains and average variance extracted (AVE) of each domain (*N* = 395)

Domains	AVE	F1	F2	F3	F4	F5
F1 (Cognition)	**0.800**	**0.894**				
F2 (Sleep)	**0.777**	0.622^**^	**0.881**			
F3 (Behavior)	**0.570**	0.636^**^	0.485^**^	**0.755**		
F4 (Emotion)	**0.778**	0.682^**^	0.625^**^	0.615^**^	**0.882**	
F5 (Obsession)	**0.741**	0.695^**^	0.655^**^	0.683^**^	0.789^**^	**0.861**

**p **≤ 0.01;  The bold values are AVEs and the square root of AVEs of each factor, and the values in bold on the diagonal are the square roots of AVE**

**Table 6:** Pearson correlation between SMPSD-RS and PHQ-9 (*N* = 30)

Domains	PHQ-9		
	Somatic Factor	Cognitive Factor	Total PHQ-9 score
**SMPSD-RS**			
Emotion	0.791**	0.689**	0.800**
Sleep	0.695**	0.605**	0.702**
Behavior	0.617**	0.561**	0.635**
Cognition	0.523**	0.648**	0.626**
Obsession	0.460**	0.646**	0.588**
Total SMPSD-RS score	0.731**	0.748**	0.795**

**p **≤ 0.01**

## Discussion

### Factor structure of the SMPSD-RS

The EFA and CFA yielded a 24-item SMPSD-RS scale covering five domains: cognition (four items), sleep (four items), behavior (five items), emotion (six items), and obsession (five items). Specifically, c*ognition* refers to changes in thinking form or speed. *Sleep* refers to changes in sleep state, e.g., a decrease in sleep time. *Behavior* refers to behaviors related to recovery or emotional changes. *Emotion* refers to a state of low or out-of-control mood. *Obsession* refers to a preoccupation with one’s own and surrounding environment. These clinically explainable symptoms of PSD can be found in stroke patients in the rehabilitation stage (within one to six months following stroke).

The five-factor structure model demonstrated good model fit indices, which confirmed the stable construct validity of the SMPSD-RS. In addition, all the item factor loadings exceeded 0.500, which indicated acceptable convergent validity of the SMPSD-RS. Besides, the five-factor structure model explained 81.673% of the variance of PSD, suggesting that the retained factors explained enough total variance of PSD in the rehabilitation stage. Furthermore, the domain variances explained by the five selected factors all exceeded 5%, indicating that each factor explained enough variance of their own domain.

### Reliability and validity of the SMPSD-RS

The Cronbach’s α and the composite reliability of the SMPSD-RS were all greater than 0.700, and the corrected item-total and item-subscale correlations of the SMPSD-RS were all greater than 0.400, which indicated that the SMPSD-RS was reliable for assessing PSD in the rehabilitation stage. In addition, the test-retest reliability of the overall measurement and domains were all acceptable, which indicated that the results of the scale were stable over time.

The acceptable AVEs confirmed the discriminant validity of the SMPSD-RS. In addition, the square root of AVEs of every counterpart individual domain was more than the correlation coefficient between domains, which further proved the good discriminant validity of the SMPSD-RS [39]. Furthermore, good concurrent criterion validity of the SMPSD-RS was evidenced by the strong and significant correlation between the SMPSD-RS and the PHQ-9. Specifically, the emotion domain of the SMPSD-RS showed the strongest correlation with the domains and total score of the PHQ-9, which indicated that the emotion domain of the SMPSD-RS may be able to measure the core symptoms of PSD during the rehabilitation stage. There was also a strong correlation between the sleep domain of the SMPSD-RS and the domains and total scores of the PHQ-9. This evidence suggested that sleep may also a significant symptom of PSD in the rehabilitation stage. In addition, the cognition domain of the SMPSD-RS was strongly correlated with the PHQ-9 cognitive domain, which suggested that the SMPSD-RS could effectively identify cognitive impairment in patients with PSD in the rehabilitation stage.

### Strength of the SMPSD-RS

Based on the PHQ-9, which is highly sensitive for PSD screening [4], the five-domain symptom measurement of PSD in the rehabilitation stage is clinically interpretable. As mentioned earlier, PSD is still an under-appreciated clinical condition, but it has been proven to be a risk factor affecting the efficacy of the rehabilitation process and the quality of life of stroke patients [10]. From this point of view, as a clinical evaluation measurement, self-evaluation of the clinical symptoms of PSD using the SMPSD-RS in the rehabilitation stage seems to be feasible.

Compared with current measures used to evaluate PSD symptoms, our instrument for screening PSD symptoms in the rehabilitation stage has significant advantages. First, compared to the DSM-V, the SMPSD-RS is quick and easy to administer (self-rated, and consisting of only 24 mood-related items). Second, the SMPSD-RS is specifically designed for stroke patients in the Chinese population, which means that it is more sensitive to this specific stroke population compared with other rating scales for general depression currently used to screen for PSD in the rehabilitation stage. Third, the SMPSD-RS has obvious advantages compared with the rating scales that are currently used specifically for PSD. For example, since participants involved in the development of the SMPSD-RS had a large age span, it avoids the influence of age on the screening results compared with the PSDRS; the SMPSD-RS is a more comprehensive screening tool for symptoms of PSD compared with the Yale-Brown Single Item Screening Question [19]; the SMPSD-RS was developed using a large sample (*n* = 780) compared with the PSDS [20]; and it was specifically developed for the time frame of one to six months after stroke compared with the ESM-PSD, which was developed for use in the acute phase (seven to thirty days following stroke) [31]. In summary, the SMPSD-RS was specifically developed to screen for PSD in the rehabilitation stage, which was proven to be reliable and valid in the current study population.

### Limitations

This study has several limitations. First, this study did not include data on specific parts or on the severity of stroke. These factors may affect the degree of depression in patients. Second, the cross-sectional design and purposive sampling procedure may affect the generalizability of the SMPSD-RS. Third, there is still a lack of predictive validity testing, which is a necessary step to evaluate the validity of clinical measurements. Finally, although the SMPSD-RS was confirmed as reliable and valid according to the excellent statistical results, future empirical research is needed to further confirm its usefulness in various clinical settings.

## Conclusions

In conclusion, the SMPSD-RS was considered to have a stable factor structure and was confirmed to be reliable and valid in assessing the symptoms of PSD in the rehabilitation stage in stroke patients. The SMPSD-RS may serve as a potential measurement to effectively screen symptoms of PSD in the rehabilitation stage, which is a basis to develop targeted interventions to improve the prognosis and quality of life of stroke patients.

## Data Availability

The datasets used and/or analyzed during the current study are available from the corresponding author on reasonable request.

## Abbreviations

PSD: Post Stroke Depression
EFA: Exploratory factor analysis
CFA: Confirmatory factor analysis
SMPSD-RS: Symptom Measurement of Post-Stroke Depression in the Rehabilitation Stage
DSM-V: Diagnostic and Statistical Manual of Mental Disorders-V
PHQ-9: Patient Health Questionnaire-9
KMO: Kaiser-Meyer-Olkin
ICC: Intraclass correlation coefficient
AVE: Average variance extracted
CFI: Comparative fit index
TLI: Tucker-Lewis index
RMSEA: Root mean square error of approximation
SRMR: Standardized root mean square residual
